# Supplementary material for: CAPA neuropeptides and their receptor form an anti-diuretic hormone signaling system in the human disease vector, Aedes aegypti
Source: Sci Rep. 2020 Feb 4;10:1755. doi: 10.1038/s41598-020-58731-y (PMC7000730; doi:10.1038/s41598-020-58731-y)
Supplement: Supplementary file 1 — SI figures captions and tables. [file 41598_2020_58731_MOESM1_ESM.docx]

*** Supporting information for: ***

­­­CAPA neuropeptides and their receptor form an anti-diuretic hormone signaling system in the human disease vector, *Aedes aegypti*

**Authors:**

Farwa Sajadi, Ali Uyuklu, Christine Paputsis, Aryan Lajevardi, Azizia Wahedi, Lindsay Taylor Ber, Andreea Matei and Jean-Paul V. Paluzzi*

Affiliation:
Department of Biology, York University, 4700 Keele Street, Toronto, Ontario, M3J 1P3, Canada.

*** Corresponding Author:**

E-mail: paluzzi@yorku.ca

Phone: (416) 736-2100 ext. 20999

Lumbers Building, Room 221

Department of Biology, York University,

4700 Keele Street, Toronto, Ontario, M3J 1P3, Canada.

**Keywords:**

CAPA; CAP2b; G protein-coupled receptor; Malpighian tubules; anti-diuresis; DH_31_; mosquito natriuretic hormone

**Supplementary file captions:**

**Figure S1.** Sequence and gene structure of *A. aegypti* anti-diuretic hormone receptor. (A) The complete cDNA sequence (lowercase) and deduced protein sequence comprised of 712 amino acid residues (uppercase) along with predicted transmembrane domains (denoted by black highlighted residues) and other features as reported in the results text. Predictions of receptor features are described in the methods section. Nucleotides belonging to different exons are indicated by alternative blue/black font colour. Predicted polyadenylation signal is underlined in the 3’ untranslated region. (B) Exons with relative size to one another drawn to scale and denotes the open reading frame (in darker gray shading) beginning with the start codon in the third exon and stop codon within the eleventh exon. Intron sizes are predicted based on comparison of the deduced completed cDNA sequence with the *A. aegypti* genome scaffolds assessed on a local database using Geneious bioinformatics software (see methods for details). Predicted intron sizes range from as small as 415bp (between exons 6-7) and as large as 74,016bp (between exons 1-2) with the entire gene spanning a genomic region of >351kb.

**Figure S2.** Molecular phylogenetic relationship of insect CAPA receptors inferred using the maximum likelihood method. Shown is the tree with the highest log likelihood with the numbers adjacent to the branches denoting the percentage of trees in which the associated taxa clustered together. A heuristic search was conducted to deduce an initial tree by applying Neighbor-Join and BioNJ algorithms to a matrix of pairwise distances estimated using a JTT model. Following this initial analysis, the topology with superior log likelihood value was selected automatically. Branch lengths are drawn to scale and denote the number of substitutions per site based on the final analysis involving 42 amino acid sequences and a total of 206 residue positions in the final data set with positions containing gaps and missing data removed. The human neuromedin U receptor 2 was included in the analysis and designated as the outgroup.

**Figure S3.** CAPA immunoreactivity observed in regions of the nervous system aside from the strongly-staining pair of neurosecretory cells in each of the abdominal ganglia. (A) CAPA immunoreactive staining in the brain showing a bilateral pair of neurons in each hemisphere of the brain and immunoreactive processes in the central margin with unknown origin. In the posterior suboesophageal ganglion, a number of small bilaterally-paired neurons (20-30 cells total) were detected. (B) In the fused thoracic ganglia, CAPA immunoreactive processes were observed on the ventral surface, with no consistently detected immunorective neurons. Although a qualitative observation, CAPA immunoreactive staining was substantially weaker in the brain, SOG and thoracic ganglia since exposure and gain settings on the fluorescence microscope were adjusted substantially to enable detection of weak immunoreactive staining. Scale bars: 100µm.

**Figure S4.** Expression analysis of CAPA neuropeptide (anti-diuretic hormone) transcript in different regions of the nervous system relative to whole adult (A) male and (B) female *A. aegypti* mosquitoes. Different letters denote bars that are significantly different from one another as determined by one-way ANOVA and Tukey’s multiple comparison post-hoc test (p < 0.01). Data represent the mean ± standard error (n = 3).

**Figure S5.** Effects of *Aedae*CAPA-1 on *in vitro* fluid secretion rates of DH_31_-stimulated MTs of adult female *A. aegypti*. Doses of 10^-6^M to 10^-17^M *Aedae*CAPA-1 were applied to MTs together wish DH_31_ for 60 minutes. Treatments that are significantly different from control MTs stimulated with DH31 alone are denoted with an asterisk, as determined by a one-way ANOVA and Bonferroni multiple comparison post-hoc test (p<0.05). Data represent the mean ± standard error (n=6-19).

**Table S1.** Oligonucleotides used for initial amplification and subsequent identification of the complete cDNA (including 5’ and 3’ UTR) encoding the *Aedes aegypti* anti-diuretic hormone (CAPA) receptor.

| Oligo name | Oligo sequence (5’3’) | Function | Accession (nucleotide position) |
| --- | --- | --- | --- |
| AedesCAPAr F0 | GTGACCATTCTCTTCACGG | amplification of partial capaR sequence | MN433886 (1326-1344) |
| AedesCAPAr R0 | CAGCTTGGAGCTCTCGCAGC | amplification of partial capaR sequence | MN433886 (2327-2308) |
| AedesCAPAr F1 | CGTCGTGGGCAATTTGATT | 3’RACE primer#1 | MN433886 (1361-1379) |
| AedesCAPAr F2 | TATCCGATTTGATCCTGCTGC | 3’RACE primer#2 | MN433886 (1450-1470) |
| AedesCAPAr F3 | GTTTCTGGCCATCTGTCATCC | 3’RACE primer#3 | MN433886 (1616-1636) |
| AedesCAPAr R1 | GAAAACAGCCACGTATTGACC | 5’RACE primer#1 | MN433886 (2128-2108) |
| AedesCAPAr R2 | TCCGGATAATCGCCTTTTTCG | 5’RACE primer#2 | MN433886 (2007-1987) |
| AedesCAPAr R3 | GATTTGCATTCCCATCCG | 5’RACE primer#3 | MN433886 (1913-1896) |

**Table S2.** List and primary structure of several insect neuropeptides tested for functional activation of the mosquito anti-diuretic hormone (CAPA) receptor using heterologous bioassay. NA denotes peptides with no detectable activity when tested up to 10μM.

| Peptide Family (Name) | Sequence | EC_50_ on CAPAr | Species (reference) |
| --- | --- | --- | --- |
| CAPA (CAPA1) | GPTVGLFAFPRV-NH_2_ | 6.76nM | *Aedes aegypti*  (Predel et al., 2010) |
| CAPA (CAPA2) | pQGLVPFPRV-NH_2_ | 5.62nM | *Aedes aegypti*  (Predel et al., 2010) |
| Pyrokinin-1 (PK1) | AGNSGANSGMWFGPRL-NH_2_ | >10μM | *Aedes aegypti*  (Predel et al., 2010) |
| Pyrokinin-2 (PK2-1) | NTVNFSPRL-NH_2_ | NA | *Rhodnius prolixus* (Paluzzi & O’Donnell, 2012) |
| Pyrokinin-2 (PK2-2) | SPPFAPRL-NH_2_ | NA | *Rhodnius prolixus* (Paluzzi & O’Donnell, 2012) |
| SIFamide peptide (SIFa) | GYRKPPFNGSIF-NH_2_ | NA | *Aedes aegypti*  (Predel et al., 2010) |
| Extended FMRFamides (FMRFa-1) | SALDKNFMRF-NH_2_ | NA | *Aedes aegypti*  (Predel et al., 2010) |
| Short neuropeptide F (sNPF) | KAVRSPSLRLRF-NH_2_ | NA | *Aedes aegypti*  (Predel et al., 2010) |
| Myoinhibitory peptide (MIP-7) | AWNSLHGGW-NH_2_ | NA | *Rhodnius prolixus*  (Paluzzi et al., 2015) |
| Leucokinin (kinin) | NSVVLGKKQRFHSWG-NH_2_ | NA | *Drosophila melanogaster* (Zandawala et al., 2018) |
| Corazonin (CRZ) | pQTFQYSRGWTN-NH_2_ | NA | *Aedes aegypti*  (Oryan et al., 2018) |

**Table S3.** Oligonucleotides used for generation of fluorescent *in situ* hybridization probes, templates for *in vitro* dsRNA synthesis and gene-specific primers for quantitative PCR of the *Aedes aegypti* anti-diuretic hormone (CAPA) receptor.

| Oligo name | Oligo sequence (5’3’) | Function | Accession (nucleotide position) |
| --- | --- | --- | --- |
| AedesCAPAF fish | GACCTGGACAGCGTCAGC | FISH probe template | XM_001650839.1 (28-45) |
| AedesCAPAR fish | CAGTTCCTTTGATCTCGGTG | FISH probe template | XM_001650839.1  (400-381) |
| AedesCAPA F1-T7 | TAATACGACTCACTATAGGGCGA… GACCTGGACAGCGTCAGC | FISH sense probe template | XM_001650839.1 (28-45) |
| AedesCAPA R1-T7 | TAATACGACTCACTATAGGGCGA… CAGTTCCTTTGATCTCGGTG | FISH anti-sense probe template | XM_001650839.1  (400-381) |
| AedesCAPA-qPCRfor | GCTGTTTGCCTTTCCAAG | qPCR forward primer | XM_001650839.1 (78-95) |
| AedesCAPA-qPCRrev | AACCACATGCCGCTGTTG | qPCR reverse primer | XM_001650839.1  (344-327) |
| AedesCAPArRNAiF1 | CCCACGGAAATCATGGACT | FISH probe and dsRNA template | MN433886 (275-293) |
| AedesCAPArRNAiR1 | GCGGATTTGCATTCCCATC | FISH probe and dsRNA template | MN433886 (1017-999) |
| AedesCAPArRNAiF-T7 | TTTAATACGACTCACTATAGGGAGACCCACGGAAATCATGGACT | FISH sense probe  and dsRNA template | MN433886 (275-293) |
| AedesCAPArRNAiR-T7 | TTTAATACGACTCACTATAGGGAGAGCGGATTTGCATTCCCATC | FISH anti-sense probe  and dsRNA template | MN433886 (1017-999) |
| AedesCAPAr-qPCRfor | GATGCTTAGCAATCCGGAA | qPCR forward primer | MN433886 (909-927) |
| AedesCAPAr-qPCRrev | GACGGAAAACAGCCACGTA | qPCR reverse primer | MN433886 (1239-1221) |
