# Supplementary material for: CAPA neuropeptides and their receptor form an anti-diuretic hormone signaling system in the human disease vector, Aedes aegypti
Source: Sci Rep. 2020 Feb 4;10:1755. doi: 10.1038/s41598-020-58731-y (PMC7000730; doi:10.1038/s41598-020-58731-y)

A

```
   1  cgaacgcgatttcagctgaagatcacttggagcagattctaatcttggggctctgtcgtgagttttgtgattttggagtgtgcgaagca
  90  aggaatcagccagaaaaaaggtagtaaatgactgcttttgtgtggaaattgttgcgctagcgtgacaaagatgatttgtgaaaaagctaa
 180  atgcatgtgtgacgcggcacggggcgtgatgtgaacatgctgccgtggtcgaccaaattgagctaactgagtgaaaagtgtttgattaat
 270  tgaggttaaccgcaacggtggattgaagtgacggttatgatgttgcagcatcgtttttatgcggaagatcttggcgcgcaacccgcaaga
 360  accgttccttggagcaaatctcagtgcagcgaaataaattagaaaaatgtgaacacagatttatcgctaatgaaaaacagttttcttatc
 450  actatttataagaggacgcaagatcgaaaagggtgcaacagggaagaaaaaaagtaataagagcgaaattggctccttgttggctgccta
 540  gaagaaaacggcggtaaagtgcatccccaatgcatttatagaaaaaacgtgaagagaagaaaagtgcatccaaagtgctggttgggaaca
 630  aaatcgattacaaaggcggcgatccattcggtcggaagtggaaaattgtttcaacgtccgtttatacaccgtttttctcaaaacttatta
 720  cggcgaaggagcggaagcaaaaaaaagaagtgatgtcggttactgggtgacgaagcacgatggttacttaaaagttggaacactcaattt
 810  cgagaacgagtaaaattttgctggcaaattccggctgcctctttgtacctgtaggacggtgtgcgaacggcgtggagtcaccaagtcagg
 900  atgactttccacaacttcgacgacgttggcagtggcacgatagaatcgtactggacaaacgccacaaccactgccaggaccacgaccacc
       M  T  F  H  N  F  D  D  V  G  S  G  T  I  E  S  Y  W  T  N  A  T  T  T  A  R  T  T  T  T   30
 990  ctcctcacagcacttctgacgacttccctcgggacgtcatcgtcggaacttggtgttgttccatcgaccgccacatccaccttgctggat
       L  L  T  A  L  L  T  T  S  L  G  T  S  S  S  E  L  G  V  V  P  S  T  A  T  S  T  L  L  D   60
1080  accggttggtggactgactacggaaatggcaccgcgctggagggatacgctggcgttagtgcgaggtggccattcaccggtgaaaactat
       T  G  W  W  T  D  Y  G  N  G  T  A  L  E  G  Y  A  G  V  S  A  R  W  P  F  T  G  E  N  Y   90
1170  tcgacccacggaaatcatggactcaccggagcggaggaaatcgtaccaccgtacgaccggtgtgatccaagaaacgaaaacttccagtgc
       S  T  H  G  N  H  G  L  T  G  A  E  E  I  V  P  P  Y  D  R  C  D  P  R  N  E  N  F  Q  C  120
1260  accgtgcaggagtttctcgagtacgcccggggaccccagcagatgccgctgtcgacagcgctgttggtgaccattctcttcacggggatc
       T  V  Q  E  F  L  E  Y  A  R  G  P  Q  Q  M  P  L  S  T  A  L  L  V  T  I  L  F  T  G  I  150
1350  ctcattaccggcgtcgtgggcaatttgattgtgtgtctggtaataattcgacatcctcagatgcacaccgccaccaactactatctgttc
       L  I  T  G  V  V  G  N  L  I  V  C  L  V  I  I  R  H  P  Q  M  H  T  A  T  N  Y  Y  L  F  180
1440  agtttggccgtatccgatttgatcctgctgctgttgggtctgccgtacgaaatcagcctctactggcaccagtacccgtacaacctgggg
       S  L  A  V  S  D  L  I  L  L  L  L  G  L  P  Y  E  I  S  L  Y  W  H  Q  Y  P  Y  N  L  G  210
1530  ttggtgttctgcaaaatgcgcgctctcatgtcggaggcatcgacttacgtgtcggtgttgacgatagtggccttttcgatggaacggttt
       L  V  F  C  K  M  R  A  L  M  S  E  A  S  T  Y  V  S  V  L  T  I  V  A  F  S  M  E  R  F  240
1620  ctggccatctgtcatccattgcacctgtacaccatgtccggattgcagcgcccggttcgcatcattgccggcctctggatcgtcagtctc
       L  A  I  C  H  P  L  H  L  Y  T  M  S  G  L  Q  R  P  V  R  I  I  A  G  L  W  I  V  S  L  270
1710  ttcagcgcagtgcctttcgccgtgttcaccgatatcgattacattctctacccaccgacccaagagaaaatcgaggactcggctttctgt
       F  S  A  V  P  F  A  V  F  T  D  I  D  Y  I  L  Y  P  P  T  Q  E  K  I  E  D  S  A  F  C  300
1800  gcgatgcttagcaatccggaaggaattcccctgtgggagctgtcgacatgcctgtttttcgccgggccgatggtggtgatgattgtactc
       A  M  L  S  N  P  E  G  I  P  L  W  E  L  S  T  C  L  F  F  A  G  P  M  V  V  M  I  V  L  330
1890  tacggccggatgggaatgcaaatccgctcccgaacgcaacgaaccgaggaactgggggtgcgaaatggttccattaacggtcctaaggta
       Y  G  R  M  G  M  Q  I  R  S  R  T  Q  R  T  E  E  L  G  V  R  N  G  S  I  N  G  P  K  V  360
1980  tctcagtcgaaaaaggcgattatccggatgctagctgccgttgtgataacgttctttgtgtgctgggcgccgtttcacgcccagaggttg
       S  Q  S  K  K  A  I  I  R  M  L  A  A  V  V  I  T  F  F  V  C  W  A  P  F  H  A  Q  R  L  390
2070  ctctttctgtacgcgcgggactggcaacacttcaacacggtcaatacgtggctgttttccgtcgcgggatggctgtactacgtttcgtgc
       L  F  L  Y  A  R  D  W  Q  H  F  N  T  V  N  T  W  L  F  S  V  A  G  W  L  Y  Y  V  S  C  420
2160  accgtcaatcccatcctgtacaacgtgatgtcccaccggtatcgggtcgctttccgggagacactttgtggccggcggcgaggctttggc
       T  V  N  P  I  L  Y  N  V  M  S  H  R  Y  R  V  A  F  R  E  T  L  C  G  R  R  R  G  F  G  450
2250  accagctttgcgcgggatcaatcgagcttccgcgaaacgacggttgacgtgaacctgggctgcgagagctccaagctgctccggctcga
       T  S  F  A  R  D  Q  S  S  F  R  E  T  T  V  D  V  N  L  G  C  E  S  S  K  L  L  R  A  R  480
2340  tcgatgatgcaatctagcaaacggtcccgctacaaaggcgctctctacacgtccaacagcgtgcgatacagtggcgaccattacatccgg
       S  M  M  Q  S  S  K  R  S  R  Y  K  G  A  L  Y  T  S  N  S  V  R  Y  S  G  D  H  Y  I  R  510
2430  cggaactccctgcagatgggcggccacattccgggaagtcgcgcctcgctctcaccaaacatgccaagcgacgtcgtggtcatgctggaa
       R  N  S  L  Q  M  G  G  H  I  P  G  S  R  A  S  L  S  P  N  M  P  S  D  V  V  V  M  L  E  540
2520  aacaagcttagcggtcgagcacgttgctacaccacatcggcctcaacgctgacgacgtccacaacgacgaccacgatgaccaccgccacc
       N  K  L  S  G  R  A  R  C  Y  T  T  S  A  S  T  L  T  T  S  T  T  T  T  T  M  T  T  A  T  570
2610  accggggaaaacatcctcaaggtacccctgataagcatcaacggtggcacgggctgtctcgtcaacaataatgtaaatagcatcaacaac
       T  G  E  N  I  L  K  V  P  L  I  S  I  N  G  G  T  G  C  L  V  N  N  N  V  N  S  I  N  N  600
2700  aactccaccaataacgccatcattcccaccgacaacagcaacaacatcaaccgtagcattagcaaggaaaacaacctttccaacgcatcc
       N  S  T  N  N  A  I  I  P  T  D  N  S  N  N  I  N  R  S  I  S  K  E  N  N  L  S  N  A  S  630
2790  accccggttccgtcttcccgggcgtcgaccagcagcctgtcaatcgaaatggcactcgataccagcagcaaacaaccgccacctaaccgg
       T  P  V  P  S  S  R  A  S  T  S  S  L  S  I  E  M  A  L  D  T  S  S  K  Q  P  P  P  N  R  660
2880  aacgacttatccctgtctgaccgaacagacagcaacgattcccaccagcaccatcctcaacagcatcccaatcctaatcatcaattaatt
       N  D  L  S  L  S  D  R  T  D  S  N  D  S  H  Q  H  H  P  Q  Q  H  P  N  P  N  H  Q  L  I  690
2970  aacggctacggaagcgataaaagcgccagtcctgctggaagtgctgtgatgcgtgagacatgtatttaacggctagcttcatcatagcag
       N  G  Y  G  S  D  K  S  A  S  P  A  G  S  A  V  M  R  E  T  C  I  *                       712
3060  ccaccaccgaggcctccgctaaacaggatgacaacgaccccaagaatagccgcatggtcagctcagtatcgaggaatgaaacccgaagcg
3150  acaagccgttaaagcttgctctgcttcaattatagatattgctgcaacgtgataagatggctcgcaacggcgtatcgtcgtcgtgccgat
3240  tagtcgatatgagtaaaaacgagcgggaaatgtcgcaatgaaaattgaaaaaaacttttacgttcaacgttccgtcatcggaatcatcga
3330  acttcaaaagcagtttttatgttcaaaactattattcgatgaaaatgtgttcactgtggttcgtttggacaatagaataaagtgatcata
3420  ttttccta(n)
```

B

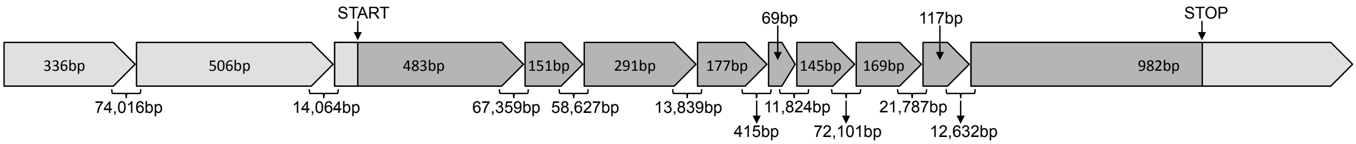

Supplement: Supplementary file 2 — SI Figure S1. [file 41598_2020_58731_MOESM2_ESM.pdf]
