# Supplementary figures and images for: CAPA neuropeptides and their receptor form an anti-diuretic hormone signaling system in the human disease vector, Aedes aegypti
Source: Sci Rep. 2020 Feb 4;10:1755. doi: 10.1038/s41598-020-58731-y (PMC7000730; doi:10.1038/s41598-020-58731-y)

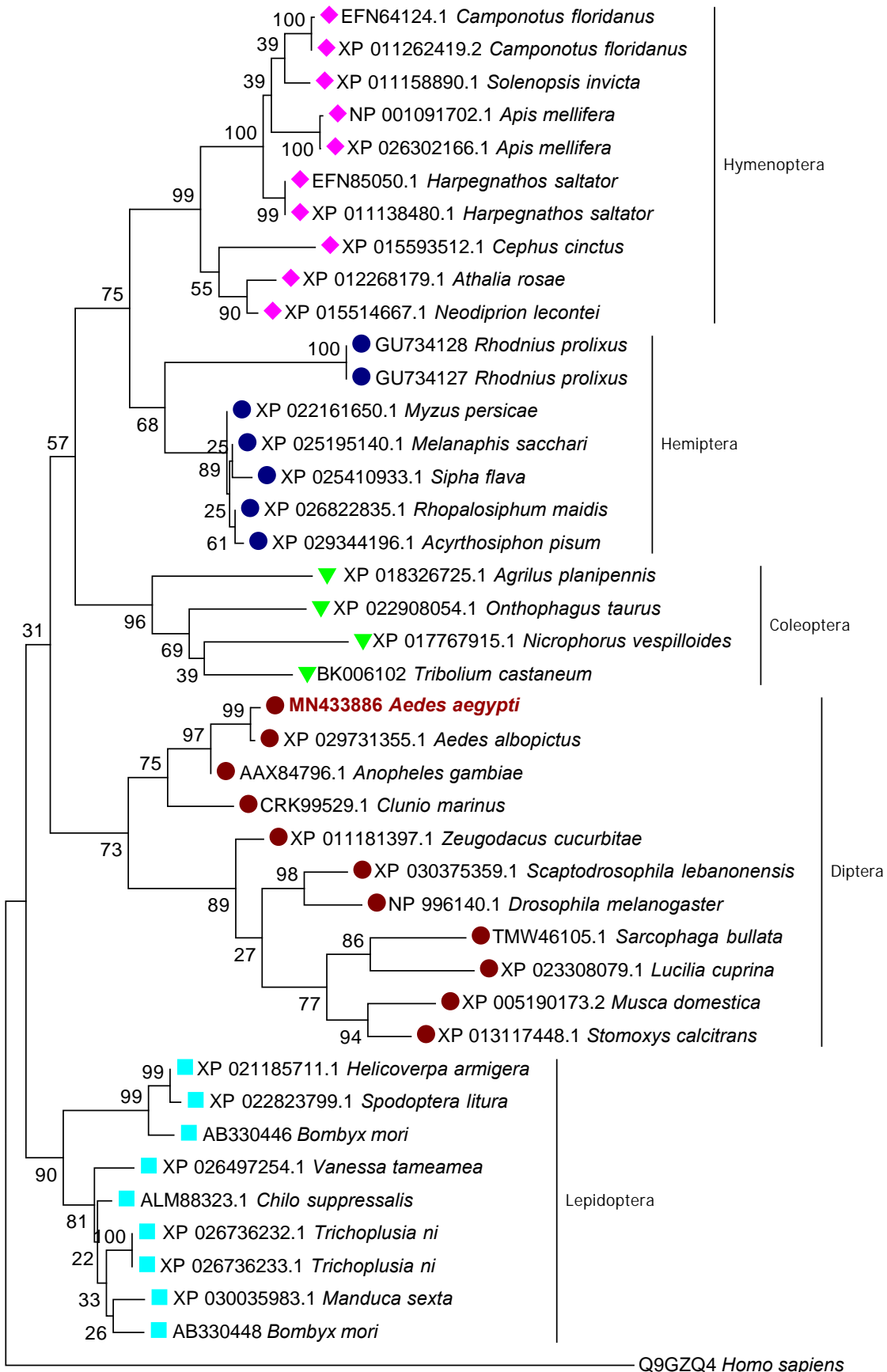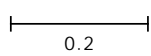

Supplement: Supplementary file 3 — SI Figure S2. [file 41598_2020_58731_MOESM3_ESM.pdf]

A

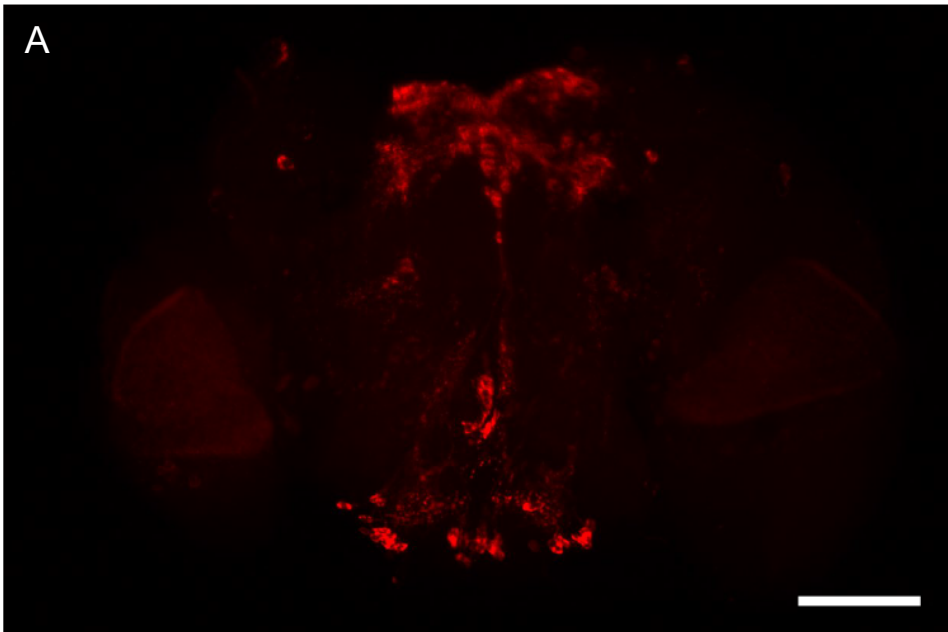

B

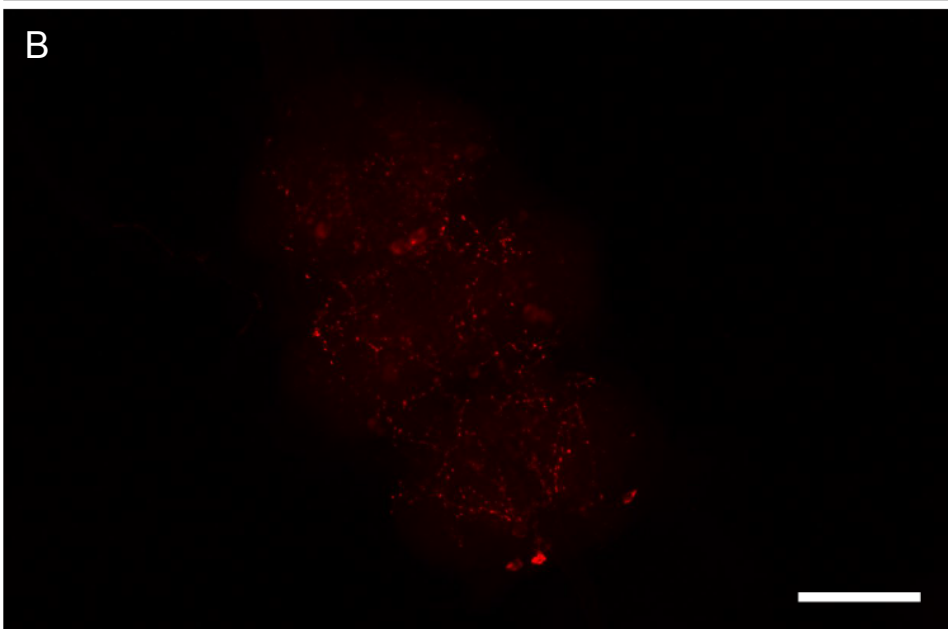

Supplement: Supplementary file 4 — SI Figure S3. [file 41598_2020_58731_MOESM4_ESM.pdf]

**A**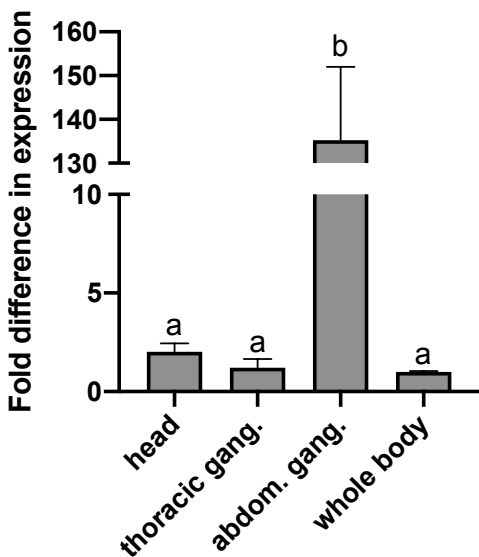**B**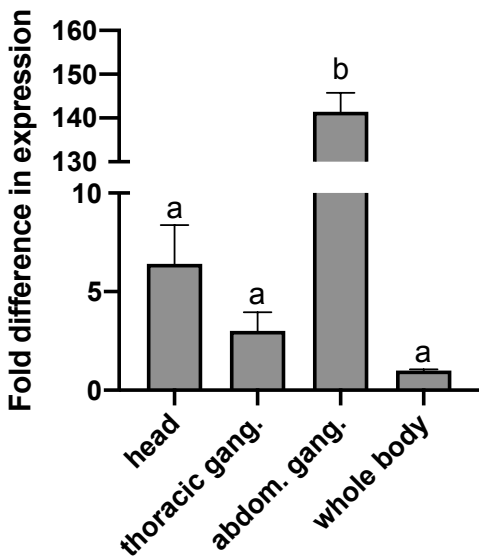

Supplement: Supplementary file 5 — SI Figure S4. [file 41598_2020_58731_MOESM5_ESM.pdf]

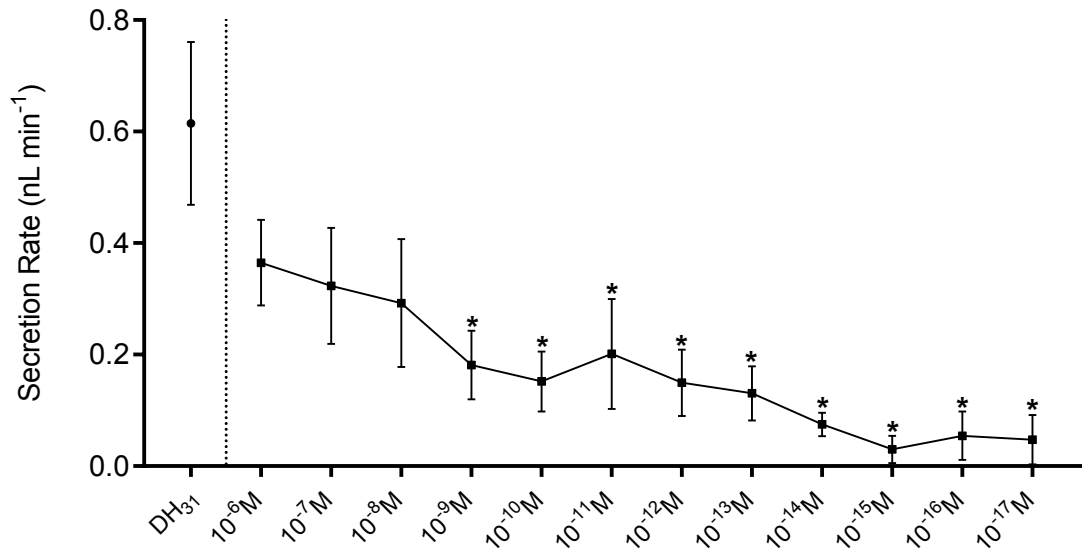

Supplement: Supplementary file 6 — SI Figure S5. [file 41598_2020_58731_MOESM6_ESM.pdf]
